# Report of Committee on Nomenclature

**Published:** 1897-04

**Authors:** 


### Report of Committee on Nomenclature.

Read before the American Dental Association, August 4th, 1896.

Your committee would report that it has endeavored to carry forward its appointed work during the past year to the best of its ability. The tabular portion of last years report which could not then be read on account of its length, has since been carefully revised and arranged.

Your committee had some difficulty in deciding upon the system of phonetic spelling to be used in indicating the sounds of vowels and syllables, but finally decided to adopt that used in the Century Dictionary, as being more exact and simpler than any other. This system is fully given at the close of this report, for purposes of reference. Your committee has also thought best to append a list of terms, the use of which should be discontinued —canine, wisdom-tooth, sixth year molar, air-chamber, odontitis, aluminium, cast, and others.

I desire to comment briefly upon a few of the features of the report, as it exhibits findings which are at variance with our common views, particularly as it relates to the pronunciation of words. You will notice, first, that the word abnormity is given preference over abnormality; it is shorter. The distinction between absorption and resorption is sharply drawn. These words are frequently employed as synonyms, but they are not synonyms. The accent in the word alloy is placed upon the last syllable. Aluminum replaces aluminium. In the word anæsthetic the diphthong æ has substituted for it the vowel e.

You will notice the plural of apex, which has its accent upon the a is apices, the first syllable being ap,–ap′ices. Proximate and proximal are preferred to approximate and approximal; the words are the stems upon which any prefixes or suffixes may be grafted.

The meeting of the masticating surfaces of the upper and lower teeth is frequently spoken of as an articulation; it is more accurately described by the word occlusion. In the word bifurcated the accent is placed upon the second, not the first syllable. The words calcareous and calcific are frequently used interchangeably: this is an error. A calcareous substance is one formed of or containing lime; a calcific body is one which forms lime. A great deal of confusion surrounds the two terms cast and model. An arbitrary distinction has been suggested, so far as dentistry is concerned, by making model to serve as the term for a plaster cast which is to be reproduced in metal; cast to be applied to the plaster not so reproduced. The practice of calling all plaster casts models appears to be more acceptable. Of the two pronunciations of cement, cement′ is preferred to cem′ent.

Contour is pronounced contour′, not con′tour. Cor′onal is correct, coro′nal incorrect. The majority of dictionaries give the preference to dentin over dentine. In fora′men the accent is upon the a′, not upon am′. The spelling gage has generally displaced gauge. In the word gingivae the accent is upon the second syllable and the i′ in this syllable is long, it is gin-ji′-vae, not gin-givae. The singular of matrix is ma′trix the plural mat′rices. Pericementum should be the term employed instead of periosteum. Sixth and twelfth-year molars are solecisms. The word solder is omitted from the list; it is to be pronounced sod-der, not sol-der. The words vulcanite and rubber, often used as synonyms, should be distinguished. Rubber, a soft body of certain composition; vulcanite, a hard body of a different chemical composition. The word obturator is restricted to to appliances which cover an opening in the hard palate. A velum is an appliance designed to remedy a deficiency of the soft palate. The terms sweating and autogenous soldering appear to be unsatisfactory.

Dr. Burchard called attention to the following points. You will notice in the list that the word abnormality is defined as the state or quality of being abnormal; irregularity, deformity, malformation. The word is given as a noun, and you will see that the definition implies, in part, an adjective. Instead of the definition given it is suggested that one founded upon the etymology of the word would be more precise and explanatory: An abnormality is an abnormal thing, not state. "It is a variation from a general typal form. It is given as a definition of alloys that they are compounds of metals produced by fusion." This is inexact; they are compounds formed when the metals are in a state of fusion. In the verb definition of the same word, to alloy, to reduce the purity of, or other-wise modify metals by admixtures, the words "with other metals" should be appended. For the purity of a metal may be reduced without forming an alloy; for instance the purity of silver is reduced by admixture with sulphor, oxygen, chlorin, bromin, and so on, and yet such compounds, it is needless to state, are not alloys. The words analgesia and analgisics are omitted. I think a distinction should be drawn between anæsthesia and analgesia. As a rule we find these words used as synonyms. Anæsthesia is a word which was coined by Dr. Oliver Wendell Holmes to designate the state of insensibility produced through the inhalation of ether or chloroform. It refers to a general abolition of sensation. Analgesa denotes explicitly an absence of pain. Analgesics would therefore include anodynes and obtundents. The root aesthesis refers to psysichical preceptions, that is, sensation in the sense that the mind perceives, therefore the title anæsthetics should be applied to agents which produce general unconsciousness; analgesices to those which destroy sensation, and do not affect general conciousness. For the word anomalous we find the definition: "Deviating from that which is natural." This defines an abnormality, not an anomaly. A definition to be in consonance with the etymology of the word, should state, "Deviating widely from that which is natural." The same adverb should be inserted in the definition of anomaly. The definition of asepsis is unsatisfactory and not precise. It states, absence of blood poisoning, exemption from putrefaction and its consequences. The word sepsis should be substituted for putrefactive.

These words are not mutually synonmous. Putrefaction always implies sepsis, but sepsis may or may not be due to putrefaction. A comprehensive definition would be, asepsis is an absence of those substances the absorption of which causes the condition of septicæmia. In the definition of antiseptics, septic should be substituted for putrefactive. In the definition of atavism, the word recurrence is used instead of the usual one, reversion, and it is perhaps better, and it more precise. In the definition of the word atrophy, it says, a gradual wasting of the body. It would be more embracive to say a wasting of a tissue, an organ, or the body, for atrophy of a single part is very common. For the definition of asphyxia, the following is submitted, in lieu of that given in the pamphlet: Asphyxia is a suspension of vitul phenomena due to a cessation of normal respiration. In the definition of calcareous, it is given, "Composed of or containing lime" Calcium salts should be substituted for lime. When we say lime we mean the oxide of calcium; calcareous may or may not refer to the oxid; in fact, it rarely does when used in connection with physiology. In defining epithelium, it is spoken of as a very delicate membrane. This is misleading, for, in point of fact, epithelium represents resistance. Modified epithelial tissue is the most resistant of all tissues. The very delicate should be omitted. The following definition is suggested: Epithelium, the external cells covering the surface of the body, lining mucous passages and glands. The word odontitis is an impossibility; we cannot have inflammation of all parts of a tooth. Odontoblasts are defined as cells which are active in the formation of dentine. Many other cells are also active. The odontoblasts are the cells through which the dentine is formed. Odontoma is spoken of as a tumor composed of dentin. Although they do contain dentine, they may also, and usually do contain other dental tissue, enamel, and cementum. The word spelt ferrule is advised to be pronounced feril. This seemed so incongruous that I looked up the etymology of the word. It is traced, first, to its French source, virole, meaning an iron ring. This is in turn from the middle Latin of viriola, a little circlet; it is a derivative of ferrum, iron which is also the etymological source of ferrel. You will notice that the oo or the l sound is present, but not any sound from which feril could be derived; so that according to etymology, the pronunciation should be fer-rool or fer-rel. In works and discussions upon dentistry, the terms prosthetic and mechanical dentistry are used as mutually synonymous. It appears to me that a distinction should be drawn between them. Prosthetic dentistry includes the planning, devising, and construction of apperatus designed for the replacement of lost dental parts, Mechanical dentistry includes the technecal operations through which these appliances are constructed. Prosthetic dentistry the architect's side; Mechanical dentistry relates solely with laboratory technology. Mechanical dentistry is included in the prosthetic, and is but part of it.

At the present time when so much attention is being directed to what is general known as "Dental Technics" your committee would recommend the abandonment of this double hybrid term and substitute for it a single word derived from pure Greek roots odontotechny as noted in the list of words printed during the past year, although the definition attached to it there, is very faulty. Pyrotechny is a good word and generally used in the arts, why then should we not use as pure and good a word to describe the purely mechanical part of our work?

When your committee's work was first undertaken it was with the simple view of improving our Nomenclature for our own benefit but its sphere has widened somewhat since, and the good results of the labor expended is shown in the fact that some medical lexicographer, having heard of our work, have shown a commendable desire to secure copies of our first report with a view to including our list of terms and definitions in there respective dictionaries. If those who can will help us by their suggestions and generous criticism, we are sure that our work will bear the the best of fruit.

Respectfully submitted

S. H. GUILFORD,
LOUIS OTTOFY,
GRANT MOLYNEAUX,
THOS. E. WEEKS,
AND OTHERS.
*Committee.*

